# The Bacterial Flagellar Type III Export Gate Complex Is a Dual Fuel Engine That Can Use Both H^+^ and Na^+^ for Flagellar Protein Export

**DOI:** 10.1371/journal.ppat.1005495

**Published:** 2016-03-04

**Authors:** Tohru Minamino, Yusuke V. Morimoto, Noritaka Hara, Phillip D. Aldridge, Keiichi Namba

**Affiliations:** 1 Graduate School of Frontier Biosciences, Osaka University, 1–3 Yamadaoka, Suita, Osaka, Japan; 2 Quantitative Biology Center, RIKEN, 6-2-3 Furuedai, Suita, Osaka, Japan; 3 Centre for Bacterial Cell Biology, Medical Sciences New Building, Newcastle University, Newcastle upon Tyne, United Kingdom; University of Utah, UNITED STATES

## Abstract

The bacterial flagellar type III export apparatus utilizes ATP and proton motive force (PMF) to transport flagellar proteins to the distal end of the growing flagellar structure for self-assembly. The transmembrane export gate complex is a H^+^–protein antiporter, of which activity is greatly augmented by an associated cytoplasmic ATPase complex. Here, we report that the export gate complex can use sodium motive force (SMF) in addition to PMF across the cytoplasmic membrane to drive protein export. Protein export was considerably reduced in the absence of the ATPase complex and a pH gradient across the membrane, but Na^+^ increased it dramatically. Phenamil, a blocker of Na^+^ translocation, inhibited protein export. Overexpression of FlhA increased the intracellular Na^+^ concentration in the presence of 100 mM NaCl but not in its absence, suggesting that FlhA acts as a Na^+^ channel. In wild-type cells, however, neither Na^+^ nor phenamil affected protein export, indicating that the Na^+^ channel activity of FlhA is suppressed by the ATPase complex. We propose that the export gate by itself is a dual fuel engine that uses both PMF and SMF for protein export and that the ATPase complex switches this dual fuel engine into a PMF-driven export machinery to become much more robust against environmental changes in external pH and Na^+^ concentration.

## Author Summary

For construction of the bacterial flagellum beyond the inner and outer membranes, the flagellar type III export apparatus transports fourteen flagellar proteins with their copy numbers ranging from a few to tens of thousands to the distal growing end of the flagellar structure. The export apparatus consists of a transmembrane export gate complex and a cytoplasmic ATPase complex. Here, we show that the export engine of the flagellar type III export apparatus is robust in maintaining its export activity against internal and external perturbations arising from genetic variations and/or environmental changes. When the cytoplasmic ATPase complex is absent, the export gate complex is able to utilize sodium motive force (SMF) across the cytoplasmic membrane as a fuel in addition to proton motive force (PMF). However, the export gate utilizes only PMF as the energy source when the ATPase complex is active. An export gate protein FlhA shows an intrinsic ion channel activity. These observations suggest that the export gate intrinsically uses both PMF and SMF for protein export and that the ATPase complex switches the export gate into a highly efficient PMF-driven export engine to become much more robust against environmental perturbations.

## Introduction

Many membrane-embedded biological nanomachines utilize proton motive force (PMF) across the membrane for their biological activities. In *Escherichia coli* and *Salmonella enterica*, PMF is utilized as the energy source for ATP synthesis, solute transport, nutrient uptake, protein transport, multidrug efflux pump and flagellar motility [1]. Alkaliphilic bacteria and hyperthermophilic bacteria utilize sodium motive force (SMF) instead of PMF [1]. The flagellar motor of *E*. *coli and Salmonella* uses H^+^ as the coupling ion to power flagellar motor rotation. In contrast, the flagellar motor of marine *Vibrio* and extremely alkalophilic *Bacillus* utilizes Na^+^ as the coupling ion instead of H^+^ [2]. It has been reported that some systems such as the melibiose permease of *E*. *coli* [3] and the flagellar motor of alkalophilic *Bacillus clausii* [4] can utilize both H^+^ and Na^+^ as their coupling ion. Interestingly, the flagellar motor of *Bacillus alcalophilus* Vedder 1934 can conduct K^+^ as well as Na^+^ [5]. Each biological system appears to have been optimized for the best use of specific ions according to the environmental conditions.

The bacterial flagellum, which is responsible for motility, is a macromolecular assembly made of about 30 different proteins and consists of the basal body rings and a tubular axial structure [6–8]. Fourteen flagellar proteins are transported through these structures by its specific export apparatus for their incorporation at the distal end of the growing flagellar structure. The export apparatus consists of a PMF-driven transmembrane export gate complex made of FlhA, FlhB, FliO, FliP, FliQ and FliR and a cytoplasmic ATPase complex consisting of FliH, FliI ATPase and FliJ [6–8]. Because the flagellar export apparatus is evolutionally related to the injectisome of pathogenic bacteria, which inject virulence effector proteins into their eukaryotic host cells for invasion, these two systems are categorized to type III secretion systems [9].

The flagellar and non-flagellar type III export apparatuses require ATP and PMF as the energy source for efficient and rapid protein export [10–15]. Because the chemical energy derived from ATP hydrolysis by the ATPase is not essential for flagellar and non-flagellar type III protein export [11, 12, 15], PMF is the primary fuel for unfolding and translocation of export substrates [10]. Since the flagellar type III export apparatus processively transports flagellar proteins to grow flagella even in the presence of the extremely low ATPase activity of FliI carrying the E211D substitution, relatively infrequent ATP hydrolysis by the cytoplasmic ATPase complex is sufficient for gate activation to start processive translocation of export substrates for efficient flagellar assembly [16]. PMF consists of two components: the electric potential difference (Δ) and the proton concentration difference (ΔpH). Δψ alone is sufficient for flagellar protein export [12] but the export gate alone, in the absence of FliH and FliI, requires the ΔpH component of PMF in addition to Δψ [13]. An increase in the ΔpH component enhances flagellar protein export in the absence of FliH and FliI [13]. D_2_O significantly reduces the rate of protein export in the absence of the FliH and FliI, also indicating that H^+^ translocation through the export gate is directly coupled with protein translocation [13]. A specific interaction between FliJ and FlhA brought about by FliH and FliI switches the export gate into a highly efficient Δψ-driven export engine [13, 17]. However, it remains unknown how and why the ΔpH component is required for the export gate to act as a H^+^–protein antiporter in the absence of the cytoplasmic ATPase complex.

To clarify the role of H^+^ in flagellar protein export, we diminished the ΔpH component of PMF and investigated the export properties of a Δ*fliH-fliI flhB(P28T)* bypass mutant whose second-site FlhB(P28T) mutation increases the export efficiency of some substrates to wild-type levels and thereby restores flagellar formation in the absence of FliH and FliI [11]. We show that the *ΔfliH-fliI flhB(P28T)* bypass mutant can use Na^+^ as the coupling ion to assemble flagella in the absence of the ΔpH component, indicating that, in addition to PMF, the export gate is powered by SMF in the absence of the cytoplasmic ATPase. We also show that FlhA has both H^+^ and Na^+^ channel activities.

## Results

### Effect of external Na^+^ concentrations on flagellar protein export at external pH 7.5

**Fig 1:**
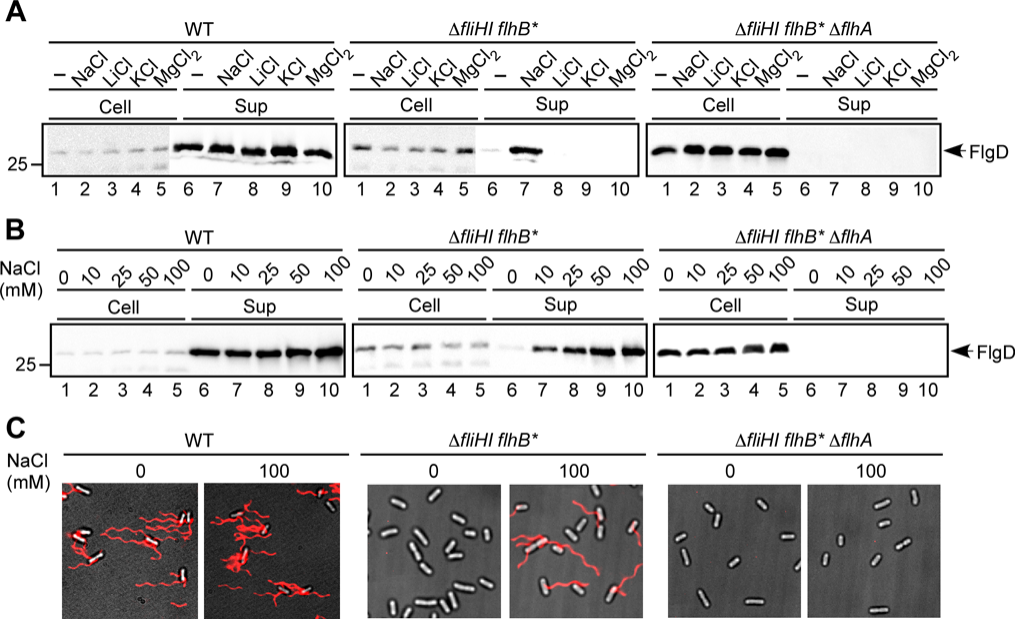
Effect of Na^+^ on flagellar protein export by the *Salmonella* wild-type (left panels), Δ*fliH-fliI flhB(P28T)* bypass mutant (middle panels) and Δ*fliH-fliI flhB(P28T)* Δ*flhA* mutant cells (right panels). (A) Effect of various cations on protein export at external pH 7.5. Immunoblotting, using polyclonal anti-FlgD antibody, of whole cell proteins (Cell) and culture supernatant fractions (Sup) prepared from SJW1103 (WT), MMHI0117 (ΔfliHI flhB*) and NH004 (ΔfliHI flhB* Δ*flhA*) grown exponentially at 30°C in T-broth containing 100 mM NaCl, 100 mM LiCl, 100 mM KCl or 100 mM MgCl_2_ at an external pH of 7.5. (B) Effect of external NaCl concentrations on the secretion level of FlgD at external pH 7.5. (C) Effect of external NaCl concentrations on flagellar filament formation. The epi-fluorescence images of the filaments labeled with Alexa Fluor 594 (red) were merged with the bright field images of the cell bodies.

Our first step was to define whether the export gate utilizes only H^+^ as the coupling ion for flagellar protein export. Our assays used a wild-type strain in which Δψ alone is sufficient for protein export and a Δ*fliH-fliI flhB(P28T)* bypass mutant that can form flagella in the absence of FliI ATPase and is known to require both the Δψ and ΔpH components for the protein export activity [11–13]. We also used an external pH of 7.5 to diminish ΔpH of the energy source because the intracellular pH is maintained at around 7.5 [13]. The growth rate of *Salmonella* cells was not affected under our experimental conditions except in no salt condition, under which it was slightly reduced compared to the presence of 100 mM NaCl (S1 Fig). In wild-type cells, neither Na^+^, Li^+^, K^+^ nor Mg^2+^ affected the secretion level of FlgD (hook cap protein) (Fig 1A, left panel). In the Δ*fliH-fliI flhB(P28T)* Δ*flhA* mutant as a negative control, no FlgD was detected in the culture supernatants (right panel). In the Δ*fliH-fliI flhB(P28T)* bypass mutant, Na^+^ dramatically enhanced FlgD secretion (middle panel, lane 7) whereas neither of Li^+^, K^+^ and Mg^2+^ did so (middle panel, lanes 8–10). The intracellular level of FlgD was not changed by these treatments (middle panel, lanes 1–5). There was no significant difference in PMF under these experimental conditions, either (S2 Fig). Consistently, the free-swimming speed, which is proportional to PMF [18], was not affected by the presence or absence of NaCl up to 100 mM (S3 Fig). The levels of FlgD secreted by Δ*fliH-fliI flhB(P28T)* showed NaCl concentration dependence at external pH 7.5 (Fig 1B, middle panel). We obtained the same results with FlgE (hook protein), FliK (hook-length control protein), FlgK (first hook-filament junction protein) and FlgL (second hook-filament junction protein) (S4 Fig). In agreement with this, more than 95% of the Δf*liH-fliI flhB(P28T)* cells had a couple of flagellar filaments in the presence of 100 mM NaCl whereas almost no flagella were observed in the absence of NaCl (Fig 1C, middle panel). We also obtained essentially the same results with an alternative Δ*fliH-fliI flhA(V404M)* bypass mutant (S5A Fig). In contrast, both the secretion levels (Fig 1B, left panel) and flagellar formation (Fig 1C, right panel) by the wild-type showed no Na^+^ dependence.

These increased levels of protein secretion and flagellar assembly with an increase in external Na^+^ concentration in the Δ*fliH-fliI flhB(P28T)* bypass mutant could be an indirect result of increased flagellar gene expression [19]. On testing flagellar promoter activities, however, the flagellar gene expression levels were slightly higher in the absence of NaCl than in its presence (S6 Fig). It has been shown that increased ionic strength facilitates the export of a flagellum-specific anti-sigma factor, FlgM, by wild-type cells, enhancing motility in soft agar [20]. Because neither Li^+^, K^+^ nor Mg^2+^ affected flagellar protein export by the Δ*fliH-fliI flhB(P28T)* bypass mutant (Fig 1A, middle panel, lanes 8–10), we suggest that Na^+^ is specific for this positive impact on flagellar protein export by the bypass mutant.

### Effect of removal of external Na^+^ on protein export by the functional gate complex

**Fig 2:**
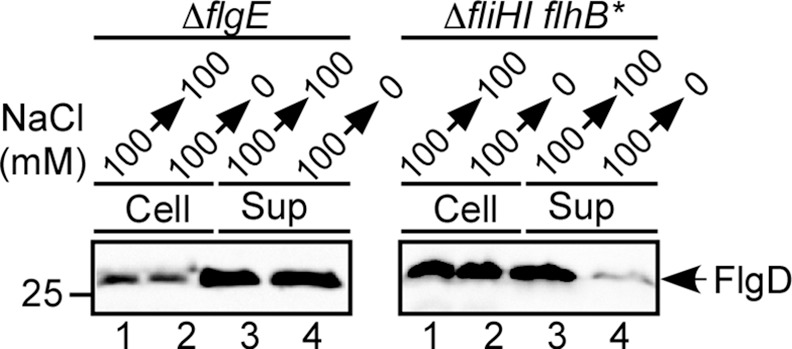
Effect of Na^+^ depletion on flagellar protein export. The level of FlgD export is shown for the wild-type export apparatus (Δ*flgE* in the left panel) and for the Δ*fliH-fliI flhB(P28T)* bypass mutant cells (right panel). The NME001 (Δ*flgE*) and MMHI0117 (ΔfliHI flhB*) cells were grown exponentially at 30°C in T-broth containing 100 mM NaCl at an external pH of 7.5. After washing twice with T-broth (pH 7.5) without 100 mM NaCl, the cells were resuspended in T-broth (pH 7.5) with or without 100 mM NaCl and incubated at 30°C for 1 hour. The whole cell (Cells) and culture supernatant fractions (Sup) were analyzed by immunoblotting with polyclonal anti-FlgD antibody.

To test whether Na^+^ directly facilitates flagellar protein export by the transmembrane export gate complex in the absence of FliH and FliI, we analyzed the effect of depletion of Na^+^ ions on protein export by the Δ*fliH-fliI flhB(P28T)* bypass mutant. We chose FlgD as a representative export substrate because the level of FlgD secretion by the bypass mutant is even higher than the wild-type level due to its poor ability to form the hook structure [11]. Since the flagellar type III export apparatus switches its export specificity from hook-type (FlgE, FlgD and FliK) to filament-type proteins (FlgM, FlgK, FlgL, FliD and FliC) upon completion of hook assembly [6–8], we used a *flgE* null mutant (Δ*flgE*) as a control; this strain continues to secrete FlgD because hook assembly does not occur and hence the export apparatus remains in the hook-type substrate specificity state. The cells were grown exponentially in T-broth (pH 7.5) containing 100 mM NaCl to produce the basal bodies with the functional type III export apparatus associated. After washing twice with T-broth (pH 7.5), the cells were resuspended in T-broth (pH 7.5) with or without 100 mM NaCl, and incubation was continued at 30°C for 1 hour. Cellular and culture supernatant fractions were prepared and analyzed by immunoblotting with polyclonal anti-FlgD antibody (Fig 2). Removal of Na^+^ ions considerably reduced the secretion level of FlgD by the Δ*fliH-fliI flhB(P28T)* bypass mutant (right panel, lane 4) but not by the Δ*flgE* mutant (left panel, lane 4). These results suggest that Na^+^ is directly involved in flagellar protein export by the export gate in the absence of FliH and FliI but not in their presence.

### Effect of the *flhB(P28T)* and *flhA(V404M)* bypass mutations on the ion selectivity of the export gate complex

To test whether the Na^+^-dependent protein export results from these bypass mutations, we analyzed the effect of Na^+^ concentration on the levels of FlgD secreted by Δ*fliH* and Δ*fliH-fliI* mutants. The FlgD secretion levels by these two mutants showed a clear dependence on external Na^+^ concentration at external pH 7.5 (S5B and S5C Fig), indicating that the *flhB(P28T) and flhA(V404M)* bypass mutations do not change the ion selectivity of the export gate complex. Therefore, we suggest that the gate can intrinsically utilize SMF in addition to PMF.

### Effect of Na^+^ channel blockers on flagellar protein export

**Fig 3:**
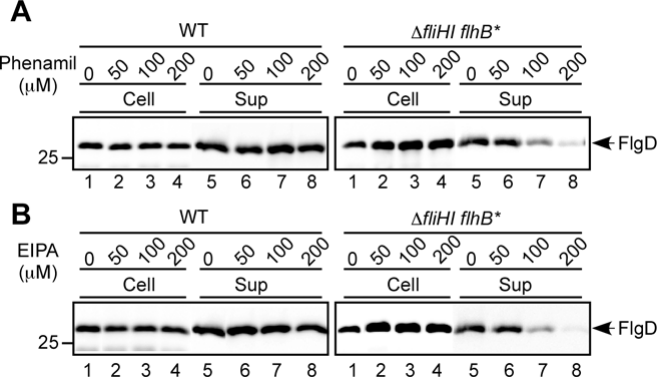
Effect of Na^+^ channel blockers on FlgD level secreted by wild-type cells (left panels) and the Δ*fliH-fliI flhB(P28T)* bypass mutant cells (right panels). Immunoblotting, using polyclonal anti-FlgD antibody, of whole cell proteins (Cells) and culture supernatant fractions (Sup) prepared from SJW1103 (WT) and MMHI0117 (ΔfliHI flhB*) grown exponentially at 30°C in T-broth containing 100 mM NaCl with 0 μM, 50 μM, 100 μM or 200 μM (A) phenamil or (B) EIPA at an external pH of 7.5.

Phenamil is known to inhibit Na^+^ channel activity without affecting cell growth [21]. The polar flagellar motor of marine *Vibrio* is powered by SMF, and the motor speed is decreased with an increase in the concentration of phenamil, showing a complete stop by 50 μM phenamil [22, 23]. To investigate whether the export gate directly utilizes Na^+^ to drive flagellar protein export, we analyzed the effect of phenamil on flagellar protein export by wild-type cells and the Δ*fliH-fliI flhB(P28T)* bypass mutant. The levels of FlgD secreted by the Δ*fliH-fliI flhB(P28T)* bypass mutant cells were markedly reduced with increasing concentrations of phenamil up to 200 μM, which was 4-fold higher than the phenamil concentration that totally inhibits the swimming motility of *Vibrio* cells (Fig 3A, right panel). The intracellular levels of FlgD were maintained. We obtained the same results with ethylisopropylamiloride (EIPA) (Fig 3B, right panel), which acts not only as an inhibitor of Na^+^/H^+^ exchange but also as a Na^+^ ion channel blocker [4,5]. Interestingly, neither phenamil nor EIPA inhibited FlgD secretion by the wild-type (left panels), indicating that the export apparatus does not use Na^+^ as the coupling ion in the presence of FliH and FliI. These treatments did not affect the swimming speeds of wild-type and *fliH-fliI* bypass mutant cells (S7 Fig), indicating that PMF was not changed at all. Therefore, we suggest that the export gate is intrinsically a dual fuel engine that can use both H^+^ and Na^+^ as the coupling ion and that the ATPase complex switches this dual fuel engine into a PMF-driven export machinery.

### Effect of external pH change on Na^+^-dependent protein export by the Δ*fliH-fliI flhB(P28T)* bypass mutant

**Fig 4:**
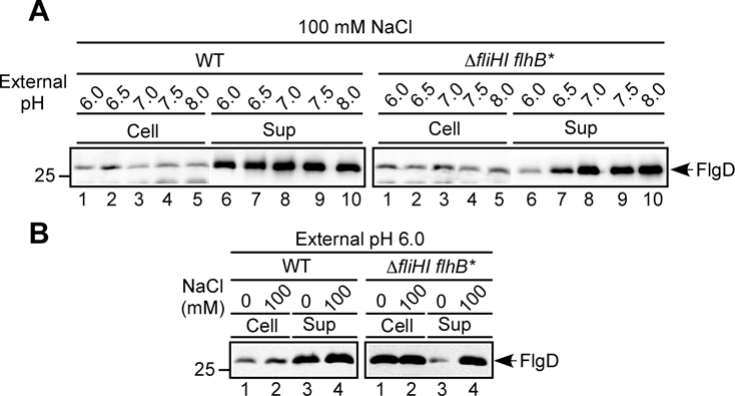
Effect of external pH change on Na^+^-dependent flagellar protein export. (A) Effect of external pH change on FlgD secretion. Immunoblotting, using polyclonal anti-FlgD antibody, of whole cell proteins (Cells) and culture supernatant fractions (Sup) prepared from SJW1103 (WT) and MMHIJ0117 (ΔfliHIJ flhB*) grown at 30°C in T-broth containing 100 mM NaCl at an external pH value of 6.0, 6.5, 7.0, 7.5 or 8.0. (B) Effect of external Na^+^ on FlgD secretion at external pH 6.0. Immunoblotting, using polyclonal anti-FlgD antibody, of whole cell proteins and culture supernatant fractions prepared from SJW1103 (WT) and MMHI0117 (ΔfliHI flhB*) grown exponentially at 30°C in T-broth with or without 100 mM NaCl at external pH 6.0.

It has been reported that the secretion level by the Δ*fliH-fliI flhB(P28T)* bypass mutant is remarkably dependent on the ΔpH component of PMF in 10 mM potassium buffer, namely in the absence of NaCl; it increases on a downward pH shift from 7.0 to 6.0 and almost diminished by an upward shift to 7.5. Since external pH change affects the ion selectivity of the stator complex of the flagellar motor of alkalophilic *Bacillus clausii*, which utilizes both H^+^ and Na^+^ as the coupling ion [4], we investigated whether external pH change influences Na^+^-dependent protein export by the Δ*fliH-fliI flhB(P28T)* bypass mutant. We varied the external pH over a range of 6.0 to 8.0 in the presence of 100 mM NaCl (Fig 4A). The level of FlgD secreted by the Δ*fliH-fliI flhB(P28T)* bypass mutant gradually increased on an upward pH shift from 6.0 to 7.0 (right panel, lanes 6–8) and then was almost constant over a range of 7.0–8.0 (lanes 8–10) although the cellular level of FlgD was not changed significantly (lanes 1–5). In wild-type cells, the secretion level of FlgD was almost constant over this pH range (left panel, lanes 6–10).

We next investigated the effect of Na^+^ concentration on FlgD secretion at external pH 6.0 (Fig 4B). The secretion level of FlgD by the Δ*fliH-fliI flhB(P28T)* bypass mutant was significantly increased by adding of 100 mM NaCl (right panel, lanes 3 and 4), indicating that Na^+^ still enhances FlgD secretion by this bypass mutant at external pH 6.0. This suggests that the transmembrane export gate complex still utilizes Na^+^ to drive flagellar protein export even when a significant pH gradient is present across the cell membrane. This raises the possibility that without FliH and FliI the export gate prefers to utilize Na^+^ rather than H^+^. In contrast, the secretion level of FlgD by the wild-type showed no Na^+^ dependence even at external pH 6.0 (left panel, lanes 3 and 4). Therefore, we suggest that FliH and FliI allow the transmembrane export gate complex to become a much more robust export engine against environmental changes.

### Effect of FliJ deletion on Na^+^-dependent flagellar protein export by the Δ*fliH-fliI flhB(P28T)* bypass mutant

**Fig 5:**
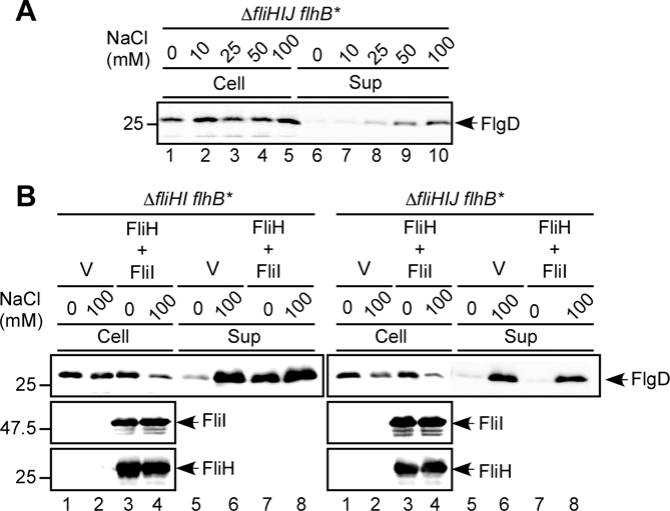
Effect of FliJ deletion on Na^+^-dependent flagellar protein export. (A) Effect of external Na^+^ concentration on FlgD secretion by the Δ*fliH-fliI-fliJ flhB(P28T)* mutant cells. Immunoblotting, using polyclonal anti-FlgD antibody, of whole cell proteins (Cells) and culture supernatant fractions (Sup) prepared from MMHIJ0117 (ΔfliHIJ flhB*) grown at 30°C in T-broth 10 mM, 25 mM, 50 mM or 100 mM NaCl at external pH 7.5. (B) Effect of external Na^+^ on FlgD secretion by the Δ*fliH-fliI flhB(P28T)* and Δ*fliH-fliI-fliJ flhB(P28T)* mutant strains in the presence and absence of FliH and FliI. Immunoblotting, using polyclonal anti-FlgD, anti-FliH or anti-FliI antibodies, of whole cell proteins and culture supernatant fractions prepared from MMHI0117 (ΔfliHI flhB*) (left panel) or MMHIJ0117 (ΔfliHIJ flhB*) carrying pTrc99A (V) or pMMHI001 (FliH + FliI) grown exponentially at 30°C in T-broth with or without 100 mM NaCl at external pH 7.5.

An interaction between FliJ and FlhA brought about by FliH and FliI is responsible for efficient PMF-driven protein export [13, 17]. Therefore, we investigated the effect of FliJ deletion on Na^+^-dependent flagellar protein export. The Na^+^ dependence of the protein export in a *ΔfliH-fliI-fliJ flhB(P28T)* mutant was not different from the Δ*fliH-fliI flhB(P28T)* strain, i.e. FlgD secretion levels increased with increasing external Na^+^ concentrations (Fig 5A). Interestingly, the Na^+^ dependence of protein export in the absence of FliJ still remained even in the presence of FliH and FliI (Fig 5B, right panel). In contrast, when FliH and FliI were expressed in the Δ*fliH-fliI flhB(P28T)* bypass mutant, there was no Na^+^ dependence (Fig 5B, left panel). This analysis confirmed that the export apparatus does not use Na^+^ for flagellar protein export in the presence of the entire ATPase complex and that FliJ is the key factor for this mechanism.

### The H^+^ and Na^+^ channel activities of FlhA

**Fig 6:**
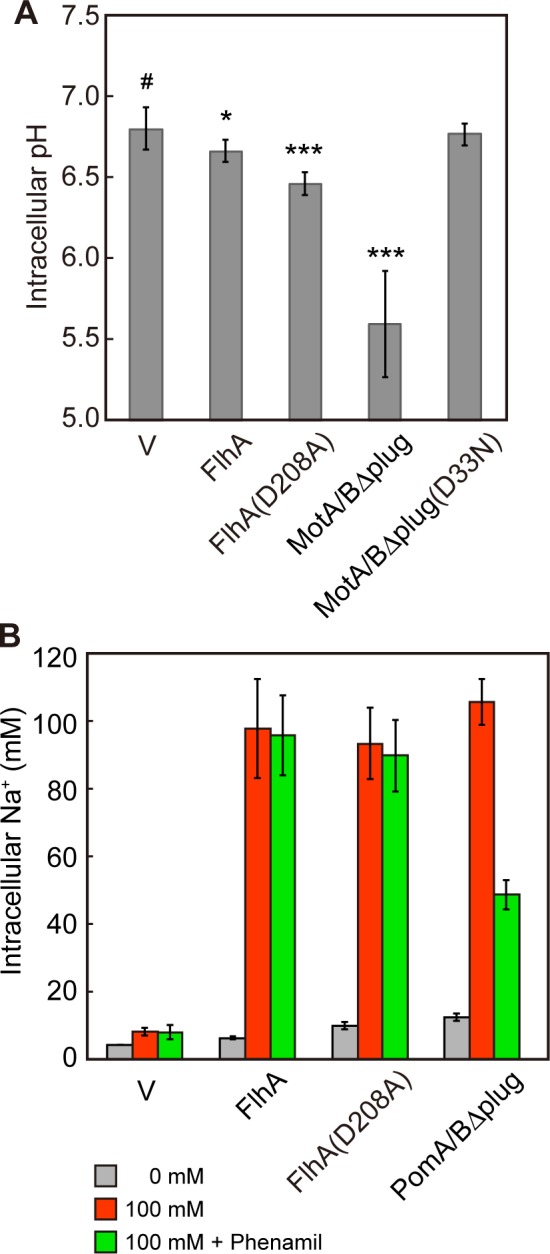
Measurements of the H^+^ and Na^+^ channel activities of FlhA. (A) Effect of overexpression of FlhA on intracellular pH change. Intracellular pH was measured with pHluorin at external pH 5.5. The BL21(DE3) strain harbouring pYC17 (pHluorin) was transformed with pBAD24 (Vector, V), pNH319 (FlhA), pYC109 (MotABΔplug) or pYC112 (MotAB(D33N)Δplug). Vertical bars indicate standard deviations of six independent biological replicates. The data that exhibited a statistically significant intracellular change compared with the vector control (#) are highlighted with an asterisk (***, *P* < 0.001; *, *P* < 0.05). (B) Effect of overexpression of FlhA on intracellular Na^+^ concentration. Intracellular Na^+^ concentration was measured with CoroNa Green in the presence and absence of 100 mM NaCl with or without 200 μM phenamil at an external pH of 7.0. The BL21(DE3) strain was transformed with pBAD24 (Vector, V), pNH319 (FlhA), pNH319(D208A) (FlhA(D208A)) or pBAD-PomΔplug (PomABΔplug). For each transformants, 200 cells were measured. Vertical bars indicate standard errors.

FlhA plays an important role in the energy transduction mechanism along with FliH, FliI and FliJ [13]. To test whether FlhA acts as an ion channel to conduct H^+^ and Na^+^, we expressed a ratiometric pH indicator probe, pHluorin [24, 25], in *E*. *coli* cells to study multicopy effect of FlhA on intracellular pH change at an external pH value of 5.5 (Fig 6A). The MotAB complex acts as a proton channel of the H^+^-driven flagellar motor, and Asp-33 of MotB is a critical proton-binding site [2]. Because a plug segment of the MotAB proton channel, consisting of residues 53 to 66 of MotB, suppresses premature proton leakage when MotAB is not assembled into the motor [26, 27], we used MotABΔplug and MotAB(D33N)Δplug as the positive and negative controls, respectively. In agreement with previous data [26, 27], the intracellular pH of the cells over-expressing MotABΔplug dropped by ca. 1.2 units in 60 min after induction with arabinose, and this intracellular pH value showed a statistically significant difference compared to that of the vector control (*P* < 0.001) using two-tailed *t*-test. The intracellular pH of the MotAB(D33N)Δplug-expressing cells was measured to be 6.77 ± 0.07, which was almost the same as the intracellular pH value of the vector control (6.80 ± 0.07). Two-tailed *t*-test revealed no significant difference between these two intracellular pH values (*P* = 0.51). Intracellular pH of the FlhA-expressing cells was 6.66 ± 0.07, which was ca. 0.1 pH unit lower than that of the vector control. This small pH drop showed a statistically significant difference compared to the vector control (*P* = 0.02).

It has been shown that a well-conserved Asp-208 of FlhA, which is located in the cytoplasmic juxtamembrane region, is essential for FlhA function. Only the conservative D208E replacement permits any function, indicating that the important feature of this residue appears to be either the negative charge of the side-chain or the ability to bind proton [28]. To test whether the FlhA(D208A) substitution suppresses such a very small decrease in the intracellular pH by over-produced FlhA, we measured the intracellular pH of the FlhA(D208A)-expressing cells. Surprisingly, the intracellular pH value dropped by ca. 0.34 units in 60 min after induction of FlhA(D208A) with arabinose, and this intracellular pH value showed a statistically significant difference compared to that of the vector control (*P* < 0.001). The expression level of FlhA(D208A) was almost the same as that of wild-type FlhA (S8 Fig). These results suggest that FlhA has an intrinsic H^+^ channel activity and that a highly conserved Asp-208 residue suppresses massive proton flow through the FlhA channel.

To test if FlhA exhibits the Na^+^ channel activity, we analyzed the effect of overproduced FlhA on intracellular Na^+^ concentration change of FlhA-expressing *E*. *coli* cells using a fluorescent Na^+^ indicator dye, CoroNa Green (Fig 6B). Because the PomAB stator complex of the marine *Vibrio* Na^+^-driven flagellar motor acts as a Na^+^ channel [2], we used PomABΔplug as a positive control. The intracellular Na^+^ concentrations of the vector control were measured to be 4.21 ± 0.04 mM and 8.03 ± 1.21 mM in the absence and presence of 100 mM NaCl, respectively. The intracellular Na^+^ concentration of the PomABΔplug-expressing cells was increased from 12.3 ± 1.0 mM to 105.7 ± 6.8 mM by adding 100 mM NaCl. These results were in good agreement with previous reports [29, 30].

Overexpression of FlhA caused a significant increment in the intracellular Na^+^ concentration in the presence of 100 mM NaCl but not in its absence. The intracellular Na^+^ concentration of the FlhA-expressing cells reached to 97.9 ± 14.7 mM, indicating that FlhA has the Na^+^ channel activity (Fig 6B). Therefore, we propose that FlhA acts as a Na^+^ channel of the export gate complex. Interestingly, the FlhA(D208A) substitution did not affect the Na^+^ channel activity of FlhA at all (Fig 6B). This raises the possibility that Asp-208 is not involved in the Na^+^ channel activity of FlhA.

The PomA(D148Y) and PomB(P16S) mutations confer the phenamil-resistant motility phenotype on *Vibrio* cells, suggesting that the phenamil-binding sites are located in both PomA and PomB [23]. We found that the level of FlgD secreted by the Δ*fliH-fliI flhB(P28T)* bypass mutant was significantly reduced by 200 μM phenamil (Fig 3), raising the possibility that the phenamil-binding site could be located in FlhA. Therefore, we analyzed the effect of phenamil on the Na^+^ channel activity of FlhA (Fig 6B). Addition of 200 μM phenamil to the PomABΔplug-expressing cells reduced the intracellular Na^+^ concentration by only about 2-fold. Since the swimming motility of *Vibrio* cells were totally inhibited by 50 μM phenamil [22,23], the binding affinity of phenamil for the PomABΔplug complex not incorporated into the *Vibrio* motor appears to be much lower than that for the PomAB complex incorporated in the motor. In contrast to the PomABΔplug complex, 200 μM phenamil did not inhibit the Na^+^ channel activity of FlhA at all. It has been shown that phenamil dissociates from the Na^+^-driven *Vibrio* motor much faster in the presence of the PomA(D148Y) and PomB(P16S) mutations than in their absence, thereby conferring the resistance to phenamil [22]. Interestingly, these two mutations are predicted to be located in the cytoplasmic juxtamembrane regions of PomA and PomB [23]. Since 200 μM phenamil did not completely inhibited the Na^+^ channel activity of the PomABΔplug complex, we suggest that the inhibitory effect of phenamil is not a direct one to the Na^+^ channel of the PomAB complex. Therefore, we propose that phenamil may not directly bind to the Na^+^ channel of FlhA to reduce the secretion activity of the export gate complex or that the binding affinity of phenamil for free FlhA may be much lower than that for FlhA incorporated into the export gate complex as seen in freely diffused PomABΔplug complex.

## Discussion

**Fig 7:**
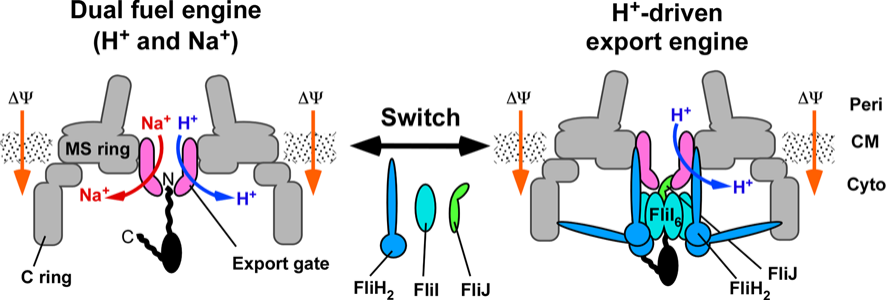
Schematic diagram of the flagellar type III export apparatus. The flagellar export apparatus is composed of a transmembrane export gate complex made of FlhA, FlhB, FliO, FliP, FliQ and FliR and a cytoplasmic ATPase complex consisting of FliH, FliI and FliJ. The export gate acts as a dual fuel H^+^/Na^+^–protein antiporter that can use both the H^+^ and Na^+^ gradients to drive flagellar protein export when the cytoplasmic ATPase consisting of FliH, FliI and FliJ cannot work properly due to internal perturbations. A specific interaction between FliJ and FlhA brought about by FliH and FliI switches a much less efficient dual fuel engine into a highly efficient H^+^-driven export engine. Δψ, membrane voltage; OM, outer membrane; PG, peptidoglycan layer; CM, cytoplasmic membrane.

PMF is the primary driving force for the flagellar and non-flagellar type III export apparatus [10]. The flagellar export gate of *S*. *enterica* is intrinsically a H^+^–protein antiporter that requires both the Δψ and ΔpH components to couple the energy of proton influx with protein export in the absence of the ATPase complex [13]. The cytoplasmic ATPase complex switches the export gate into a highly efficient, Δψ-driven protein export apparatus, and an interaction between FliJ and FlhA is key in driving this switch [13]. In this study, we showed that, in addition to PMF, the export gate can use SMF to drive flagellar protein export over an external pH range of 6.0–8.0 in the absence of FliH, FliI and FliJ (Figs 1, 4 and 5). This suggests that without FliH, FliI and FliJ the export gate alone is a dual fuel export engine that can exploit both H^+^ and Na^+^ as the coupling ion (Fig 7). Interestingly, environmental changes significantly affected flagellar protein export by the Δ*fliH-fliI flhB(P28T)* but not that by wild-type cells (Figs 1 and 4). Therefore, we propose that the export apparatus is robust and has evolved to be able to maintain protein export activity against internal or external, genetic or environmental perturbations. To achieve this level of robustness the export gate has evolved to exploit both H^+^ and Na^+^ as the coupling ion rather than becoming an exclusive PMF or SMF dependent machine.

FlhA, which consists of an N-terminal integral membrane domain with eight predicted transmembrane helices (FlhA_TM_) and a C-terminal cytoplasmic domain (FlhA_C_) [31], forms a nonameric ring structure in the export apparatus [32, 33]. FlhA_C_ not only acts as a docking platform for FliH, FliI, FliJ, export substrates and chaperone-export substrate complexes [13, 34–38] but also plays important roles in the energy coupling mechanism of flagellar type III protein export [13,17]. In this study, we showed that overexpression of FlhA resulted in a significant increment in the intracellular Na^+^ concentrations as seen in the PomAB Na^+^ ion channel complex, which works as the stator of the Na^+^-driven flagellar motor of marine *Vibrio* (Fig 6B). However, when FlhA was overproduced, only a very small decrease in intracellular pH was observed in the FlhA-overexpressing cells (Fig 6A). If overexpression of FlhA non-specifically perturbed the cell membrane, both H^+^ and Na^+^ would have leaked into the cell through the membrane, thereby increasing the intracellular concentrations of both H^+^ and Na^+^ considerably. Therefore, we conclude that FlhA has an intrinsic Na^+^ channel activity. Interestingly, neither Na^+^ nor Na^+^ channel blockers affected protein export by wild-type cells (Figs 2 and 3), indicating that the Na^+^ channel of FlhA is kept in a closed state in the presence of FliH, FliI and FliJ. Therefore, we propose that the intrinsic Na^+^ channel activity of FlhA may provide the cell with a genetic backup to rapidly compensate the occasional loss or inactivation of the ATPase complex during flagellar assembly.

A highly conserved Asp-208 of FlhA is essential for PMF-driven flagellar protein export [28]. The FlhA(D208A) substitution results in a loss-of-function phenotype [28]. Here, we found that the intracellular pH decreased by about 0.34 units in 60 min after induction of FlhA(D208A) with arabinose whereas the intracellular pH of the cells expressing wild-type FlhA decreased by about 0.1 unit (Fig 6A). The D208A mutation did not affect the expression level of FlhA at all (S8 Fig). These results indicate that overexpression of FlhA(D208A) causes massive proton leakage through its proton channel, thereby inhibiting cell growth. Therefore, we propose that FlhA also has the intrinsic ability to conduct H^+^. Since Asp-208 of FlhA is predicted to be located in the cytoplasmic juxtamembrane region [28], we propose that this Asp residue plays a regulatory role in coordinated proton flow through the FlhA proton channel coupled with protein export. Interestingly, the D208A did not affect the Na^+^ channel activity of FlhA at all (Fig 6B), raising the possibility that the Na^+^ pathway in FlhA could be distinct from the H^+^ pathway.

Based on all available information, we propose that FlhA is an energy transducer of the export apparatus for flagellar protein export. In the absence of FliH, FliI and FliJ, Na^+^ ions still showed a positive impact on flagellar protein export by the export gate even at an external pH value as low as 6.0 (Fig 4B). Although there is a significant pH gradient across the cytoplasmic membrane under this condition, the export gate prefers to use the Na^+^ gradient over the H^+^ gradient. This could explain why the *ΔfliH-fliI flhB(P28T)* bypass mutant requires the ΔpH component for flagellar protein export in addition to Δψ and why depletion of the ΔpH component and D_2_O significantly reduce the rate of protein export by this bypass mutant [13]. In the presence of FliH, FliI and FliJ, the export gate used only PMF, suggesting that the Na^+^ channel of FlhA is closed by the binding of the cytoplasmic ATPase complex to the gate. Because the intrinsic H^+^ channel activity of FlhA is quite low (Fig 6A), we propose that the cytoplasmic ATPase complex may allow FlhA to conduct H^+^ more efficiently so that proton influx is not limiting the rate of protein export. FliI is the ATPase of the export apparatus [39] and forms a homo-hexamer to exert its ATPase activity [40]. FliJ binds to the center of the FliI_6_ ring to form the FliI_6_FliJ ring, which is structurally similar to F-type and V-type ATPases [41]. FliH connects the FliI_6_FliJ ring with the export gate complex through an interaction of FliH and FlhA [42]. ATP hydrolysis by FliI ATPase activates the export gate through an interaction between FliJ and FlhA, allowing the gate to transport flagellar proteins in a PMF-dependent manner [13, 16, 17]. Therefore, we propose that FliJ acts as a switch of the energy transducer to change the ion channel properties of FlhA from a dual ion channel mode to a H^+^ channel mode (Fig 7).

## Materials and Methods

### Bacteria, plasmids and media

*Salmonella* strains and plasmids used in this study are listed in Table 1. T-broth (TB) contained 1% Bacto tryptone, 10 mM potassium phosphate pH 7.5. Ampicillin and chloramphenicol were added at a final concentration of 100 μg/ml and 30 μg/ml, respectively, if needed.

**Table 1:** Strains and plasmids used in this study.

Strains and Plasmids	Relevant characteristics	Source or reference
*E*. *coli*		
BL21(DE3)	Overexpression of proteins	Novagen
*Salmonella*		
SJW1103	Wild type for motility and chemotaxis	[47]
SJW1368	Δ*cheW-flhD*	[48]
MKM11	Δ*fliH*	[49]
MMHI001	Δ*fliH*-*fliI*	[50]
MMHI0117	Δ*fliH*-*fliI flhB*(P28T)	[11]
MMHI0132	Δ*fliH*-*fliI flhA*(V404M)	[11]
MMHIJ0117	Δ*fliH*-*fliI-fliJ flhB*(P28T)	[13]
NME001	Δ*flgE*	[51]
NH004	Δ*fliH*-*fliI flhB*(P28T) Δ*flhA*	[28]
Plasmids		
pTrc99A	Expression vector	GE Healthcare
pBAD24	Expression vector	[52]
pMMHI001	pTrc99AFF4/FliH + FliI	[11]
pNH319	pBAD24/N-His-FLAG-FlhA	This study
pNH319(D208A)	pBAD24/N-His-FLAG-FlhA(D208A)	This study
pRG19::*cat*	P_*motA*_::*luxCDABE*, Cm^r^	[46]
pRG39::*cat*	P_*fliC*_::*luxCDABE*, Cm^r^	[46]
pRG51::*cat*	P_*flgA*_::*luxCDABE*, Cm^r^	[46]
pRG53::*cat*	P_*fliE*_::*luxCDABE*, Cm^r^	[46]
pYC17	pACTrc/pHluorin	This study
pYC109	pBAD24/MotA+MotB(Δ52–71)	[27]
pYC112	pBAD24/MotA+MotB(D33N/Δ52–71)	[53]
pYVM001	pKK223-3/pHluorin(M153R)	[25]
pBAD-PomΔplug	pBAD24/PomA+PomB(Δ41–120)	M. Homma

### Secretion assay

The cells were grown with shaking in 5 ml of TB with or without various concentrations of NaCl, LiCl, KCl or MgCl_2_ at 30°C until the cell density had reached an OD_600_ of ca. 1.4–1.6. To see the effect of removal of Na^+^ on Na^+^-dependent protein export by the *ΔfliH-fliI flhB(P28T)* mutant cells, the cells were grown with shaking in 3 ml of TB (pH 7.5) with or without 100 mM NaCl at 30°C until the cell density had reached an OD_600_ of ca. 0.8–1.0. After washing twice with TB (pH 7.5), the cells were resuspended in 3 ml TB with or without 100 mM NaCl and then incubated at 30°C for 1 hour. To test the effects of phenamil and EIPA on flagellar protein export, the cells were grown with shaking in 5 ml of TB containing 100 mM NaCl at 30°C until the cell density had reached an OD_600_ of ca. 1.0–1.2. After washing the cells twice with TB containing 100 mM NaCl, the cells were resuspended in the 5 ml TB with 100 mM NaCl in the presence of various concentrations of phenamil or EIPA and incubated at 30°C for 1 hour. Cultures were centrifuged to obtain cell pellets and culture supernatants. Cell pellets were resuspended in the SDS-loading buffer, normalized to a cell density to give a constant amount of cells. Proteins in the culture supernatants were precipitated by 10% trichloroacetic acid, suspended in the Tris/SDS loading buffer and heated at 95°C for 3 min. After SDS-PAGE, immunoblotting with polyclonal anti-FlgD, anti-FlgE, anti-FliK, anti-FlgK or anti-FlgL antibody was carried out as described before [43]. Detection was performed with an ECL plus immunoblotting detection kit (GE Healthcare). At least three independent experiments were carried out.

### Measurements of free-swimming speed of motile *Salmonella* cells in liquid media

Overnight culture of *Salmonella* cells was inoculated into fresh TB with 100 mM NaCl and incubated at 30°C with shaking for 4 hours. The cells were washed twice with TB and resuspended in TB with or without various concentrations of NaCl, LiCl, KCl or MgCl_2_. To test the effects of phenamil and EIPA on free-swimming motility, the cells were resuspended in TB containing 100 mM NaCl in the presence of various concentrations of phenamil or EIPA. The swimming speed of individual motile cells was measured under a phase contrast microscopy at room temperature as described before [44].

### Observation of flagellar filaments with a fluorescent dye

The flagellar filaments produced by *Salmonella* cells were labelled using polyclonal anti-FliC antibody and anti-rabbit IgG conjugated with Alexa Fluor 594 (Invitrogen) as described [16]. The cells were observed by fluorescence microscopy as described previously [45]. Fluorescence images were analysed using ImageJ software version 1.48 (National Institutes of Health).

### Measurements of the membrane potential and intracellular pH

The membrane potential was measured using tetramethylrhodamine methyl ester (Invitrogen) as described before [13]. Intracellular pH measurements with a ratiometric fluorescent pH indicator protein, pHluorin [24, 25], were carried out as described before [27].

### Measurements of flagellar class2 and class 3 promoter activities

*Salmonella* SJW1103 and MMHI0117 strains were transformed with the pRGXX::*cat* series [46]. The cells were grown with shaking in 5 ml of T-broth with or without 100 mM NaCl at 30°C until the cell density had reached an OD_600_ of ca. 1.0–1.2. The cultures were then pipetted (200 μl) into a 96–well microplate (Greiner Bio-One). Bioluminescence and absorbance of cultures were measured using 2030 ARVO X microplate reader (Perkin Elmer) at 30°C. All microplate assays were repeated four times. Promoter activities were calculated as the value for bioluminescence intensities divided by absorbance value after background correction.

### Intracellular sodium ion measurement using CoroNa Green

The *E*. *coli* BL21(DE3) strain was transformed with a pBAD24-based plasmid. The resulting transformants were grown in TB (pH 7.0) at 30°C for 4 hours. The protein expression was induced by addition of 0.2% arabinose. After 1 h, the cells were washed three times with TB, resuspended in TB (pH 7.0) containing 40 μM CoroNa Green (Invitrogen) and 10 mM EDTA and incubated in the dark room for 60 min at room temperature. Then, the cells were washed three times with TB to remove excess CoroNa Green and resuspended in TB with or without 100 mM NaCl. To observe epi-fluorescence images, we used an inverted fluorescence microscope (IX-73, Olympus) with a 100× oil immersion objective lens (UPLSAPO100XO, NA 1.4, Olympus) and an sCMOS camera (Zyla4.2, Andor Technology). Epi-fluorescence of CoroNa Green was excited by a 130 W mercury light source system (U-HGLGPS, Olympus) with a fluorescence mirror unit U-FGFP (Excitation BP 460–480; Emission BP 495–540, Olympus). Fluorescence images of CoroNa Green were captured at every 100 msec exposure. Fluorescence image processing was performed with the ImageJ version 1.48 software (National Institutes of Health). To quantify the fluorescence intensity of each cell, integral fluorescence of CoroNa Green was measured and then the intensity of a nearby cell-less region was subtracted as the background intensity. To calibrate the intracellular sodium concentration, fluorescence intensity of the cells with CoroNa Green were measured at various sodium concentrations in TB containing 20 μM gramicidin and 5 μM carbonyl cyanide 3-chlorophenylhydrazone (CCCP) as described before [30]. All experiments were performed at 23°C.

### Statistical analysis

Statistical analyses were done using StatPlus::mac software (AnalystSoft). Comparisons were performed using a two-tailed Student’s *t*-test. A *P* value of < 0.05 was considered to be statistically significant difference. *, *P* < 0.05; **, *P* < 0.01; ***, *P* < 0.001.

## Supporting Information

S1 Fig Effect of external NaCl concentration on growth of wild-type cells and a Δ*fliH-fliI flhB(P28T)* bypass mutant. (A) SJW1103 (WT) and (B) MMHI0117 (ΔfliHI flhB*) grown at 30°C in T-broth with or without 100 mM NaCl at external pH 7.5. The OD_600_ of cultures was monitored. These data are the average of three independent biological replicates. The experimental errors are within a few %.
(TIF)

S2 Fig Measurements of total proton motive force (PMF). (A) Effect of various cations on total PMF of SJW1103 (WT) and MMHI0117 (ΔfliHI flhB*) grown exponentially at 30°C in T-broth containing 100 mM NaCl, 100 mM LiCl, 100 mM KCl or 100 mM MgCl_2_ at an external pH of 7.5. The membrane potential was measured using tetramethylrhodamine methyl ester. More than 100 cells were measured. Intracellular pH was measured with pHluorin(M153R). Six independent experiments were carried out. Vertical bars indicate standard deviations. (B) Effect of external NaCl concentrations on total PMF of SJW1103 and MMHI0117.
(TIF)

S3 Fig Effect of Na^+^ on free-swimming motility in liquid media. (A) Effect of various cations on swimming speed of SJW1103 (WT) and MMHI0117 (ΔfliHI flhB*). Swimming speeds of SJW1103 and MMHI0117 were measured in T-broth containing 100 mM NaCl, 100 mM KCl, 100 mM LiCl or 100 mM MgCl_2_ at an external pH of 7.5. More than 30 cells were measured. Vertical bars indicate standard deviations. (B) Effect of external NaCl concentrations on swimming speed of SJW1103 (WT) and MMHI0117 (ΔfliHI flhB*) at external pH 7.5.
(TIF)

S4 Fig Effect of Na^+^ on the levels of FlgE, FliK, FlgK and FlgL secreted by the wild-type, Δ*fliH-fliI flhB(P28T)* bypass mutant and Δ*fliH-fliI flhB(P28T)* Δ*flhA* mutant cells. Immunoblotting, using polyclonal anti-FlgE (1st row), anti-FliK (2nd row), anti-FlgK (3rd row) or anti-FlgL (4th row) antibody, of whole cell proteins (Cell) and culture supernatant fractions (Sup) prepared from SJW1103 (WT), MMHI0117 (ΔfliHI flhB*) and NH004 (ΔfliHI flhB* Δ*flhA*) grown exponentially at 30°C in T-broth with or without 100 mM NaCl at external pH 7.5.
(TIF)

S5 Fig Effect of external Na^+^ concentration on FlgD secretion in the absence of FliH and FliI. Immunoblotting, using polyclonal anti-FlgD antibody, of whole cell proteins (Cell) and culture supernatant fractions (Sup) prepared from (A) MMHI0132 (ΔfliHI flhA*), (B) MKM11 (Δ*fliH*), and (C) MMHI001 (Δ*fliHI*) grown at 30°C in T-broth containing 10 mM, 25 mM, 50 mM or 100 mM NaCl at external pH 7.5.
(TIF)

S6 Fig Effect of external Na^+^ on flagellar gene expression. SJW1103 (WT) and MMHI0117 (ΔfliHI flhB*) were transformed with pRG19::*cat* (P_*motA*_), pRG39::*cat* (P_*fliC*_), pRG51::*cat* (P_*flgA*_) or pRG19::*cat* (P_*fliE*_). Bioluminescence was measured as a promoter activity by a microplate reader. Vertical bars show standard deviations of four independent biological replicates.
(TIF)

S7 Fig Effect of Phenamil and EIPA on free-swimming motility in liquid media. Swimming speeds of SJW1103 (WT) and MMHI0117 (ΔfliHI flhB*) were measured in T-broth containing 100 μM phenamil or 100 μM EIPA at an external pH of 7.5. More than 30 cells were measured. Vertical bars indicate standard deviations.
(TIF)

S8 Fig Effect of the FlhA(D208A) mutation on the cellular level of FlhA. Immunoblotting, using polyclonal anti-FlhA antibody, of whole cell proteins prepared from SJW1368 carrying pBAD24 (V), pNH319 (WT) or pNH319(D208A).
(TIF)
