# Supplementary figures and images for: The Bacterial Flagellar Type III Export Gate Complex Is a Dual Fuel Engine That Can Use Both H+ and Na+ for Flagellar Protein Export
Source: PLoS Pathog. 2016 Mar 4;12(3):e1005495. doi: 10.1371/journal.ppat.1005495 (PMC4778876; doi:10.1371/journal.ppat.1005495)

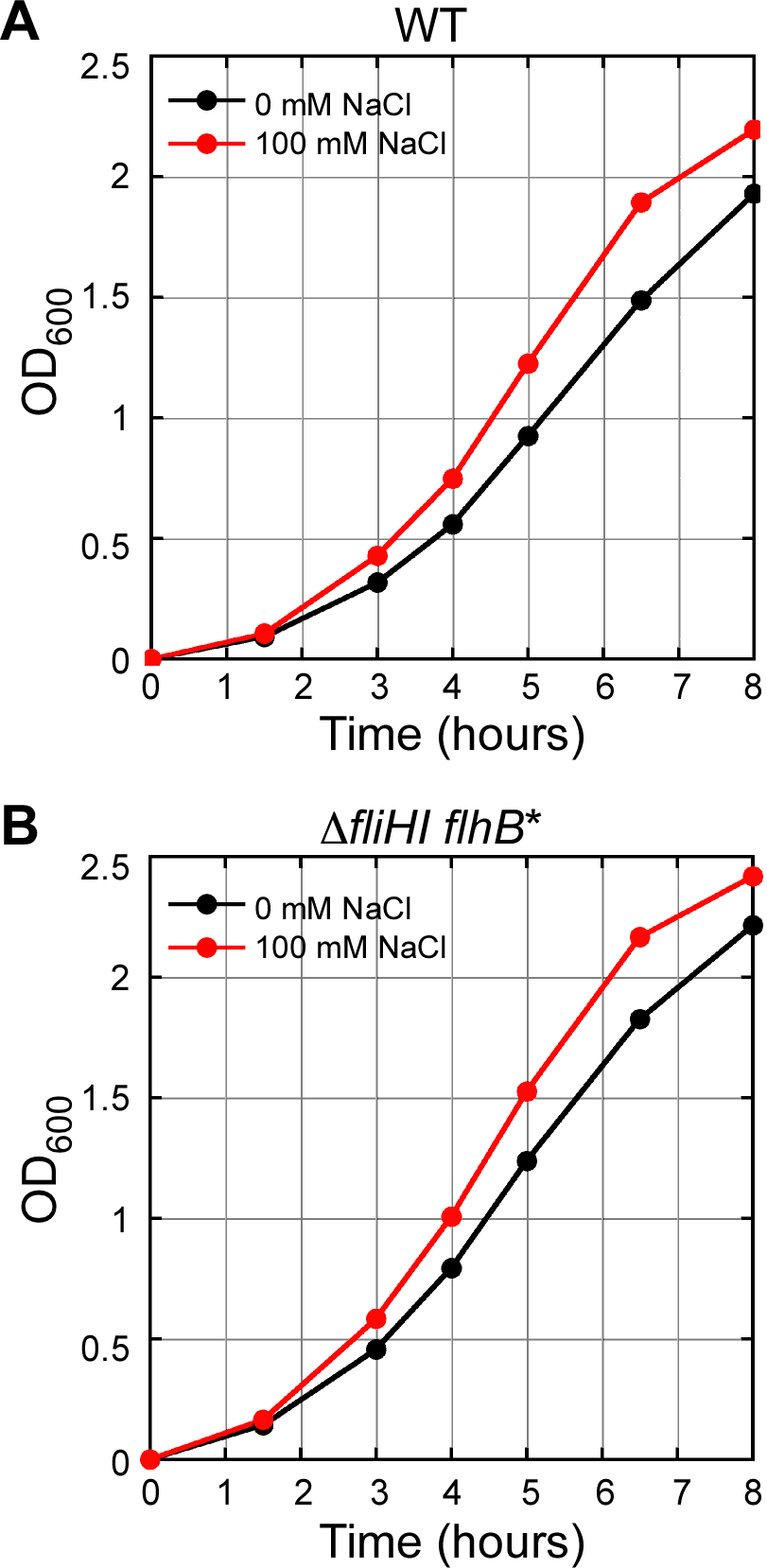

Supplement: S1 Fig — (A) SJW1103 (WT) and (B) MMHI0117 (ΔfliHI flhB*) grown at 30°C in T-broth with or without 100 mM NaCl at external pH 7.5. The OD600 of cultures was monitored. These data are the average of three independent biological replicates. The experimental errors are within a few %. (TIF) [file ppat.1005495.s001.tif]

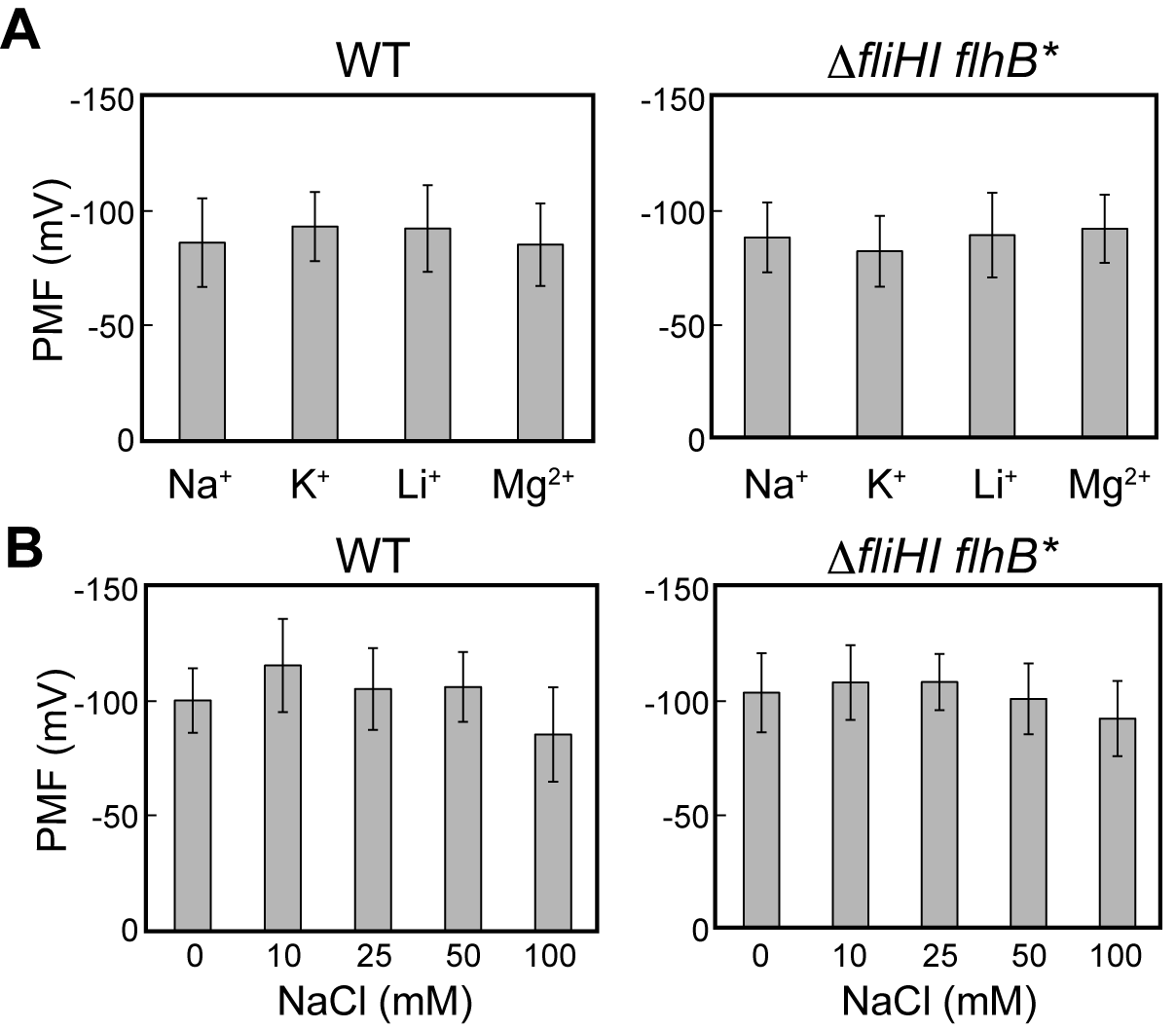

Supplement: S2 Fig — (A) Effect of various cations on total PMF of SJW1103 (WT) and MMHI0117 (ΔfliHI flhB*) grown exponentially at 30°C in T-broth containing 100 mM NaCl, 100 mM LiCl, 100 mM KCl or 100 mM MgCl2 at an external pH of 7.5. The membrane potential was measured using tetramethylrhodamine methyl ester. More than 100 cells were measured. Intracellular pH was measured with pHluorin(M153R). Six independent experiments were carried out. Vertical bars indicate standard deviations. (B) Effect of external NaCl concentrations on total PMF of SJW1103 and MMHI0117. (TIF) [file ppat.1005495.s002.tif]

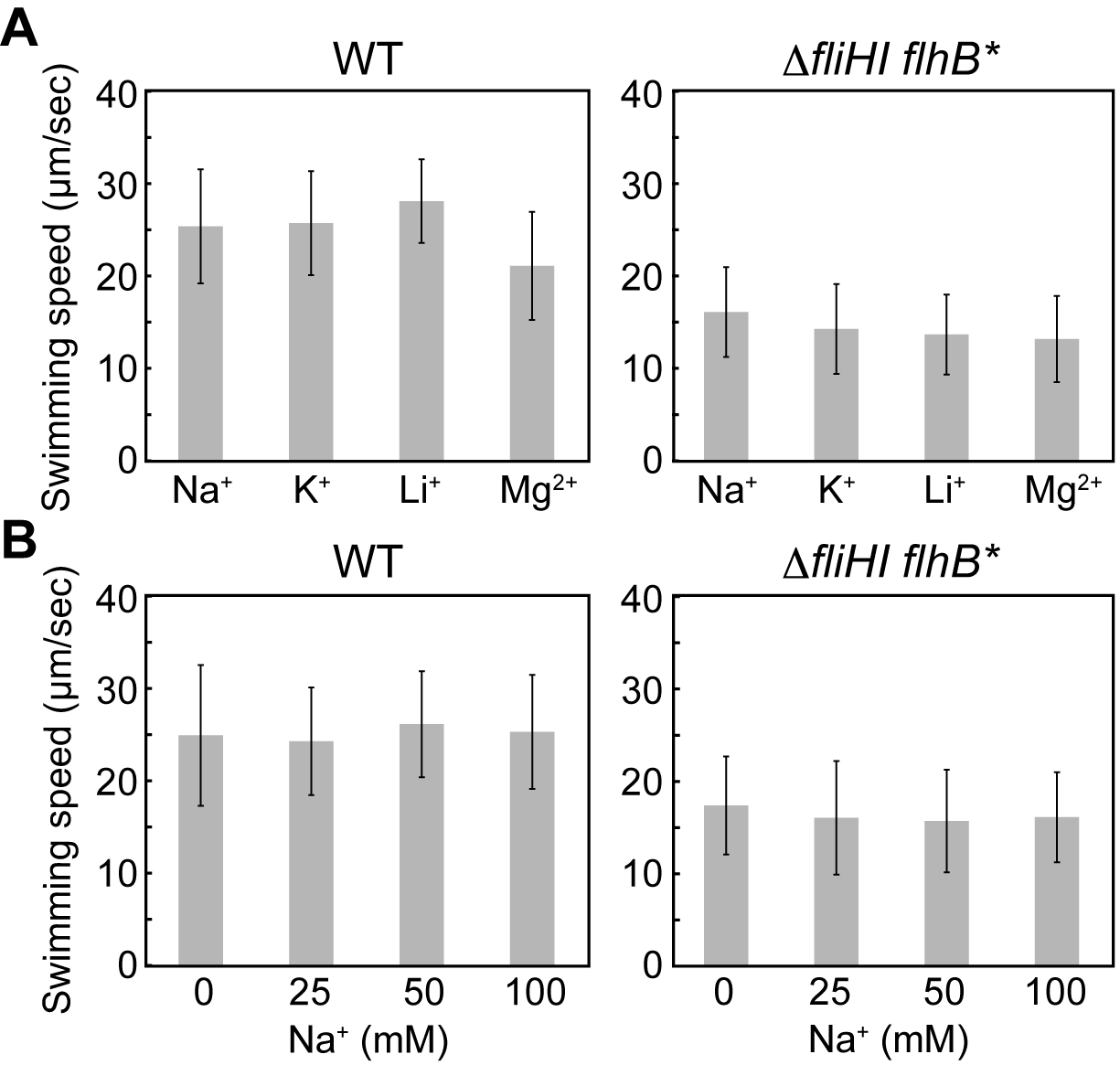

Supplement: S3 Fig — (A) Effect of various cations on swimming speed of SJW1103 (WT) and MMHI0117 (ΔfliHI flhB*). Swimming speeds of SJW1103 and MMHI0117 were measured in T-broth containing 100 mM NaCl, 100 mM KCl, 100 mM LiCl or 100 mM MgCl2 at an external pH of 7.5. More than 30 cells were measured. Vertical bars indicate standard deviations. (B) Effect of external NaCl concentrations on swimming speed of SJW1103 (WT) and MMHI0117 (ΔfliHI flhB*) at external pH 7.5. (TIF) [file ppat.1005495.s003.tif]

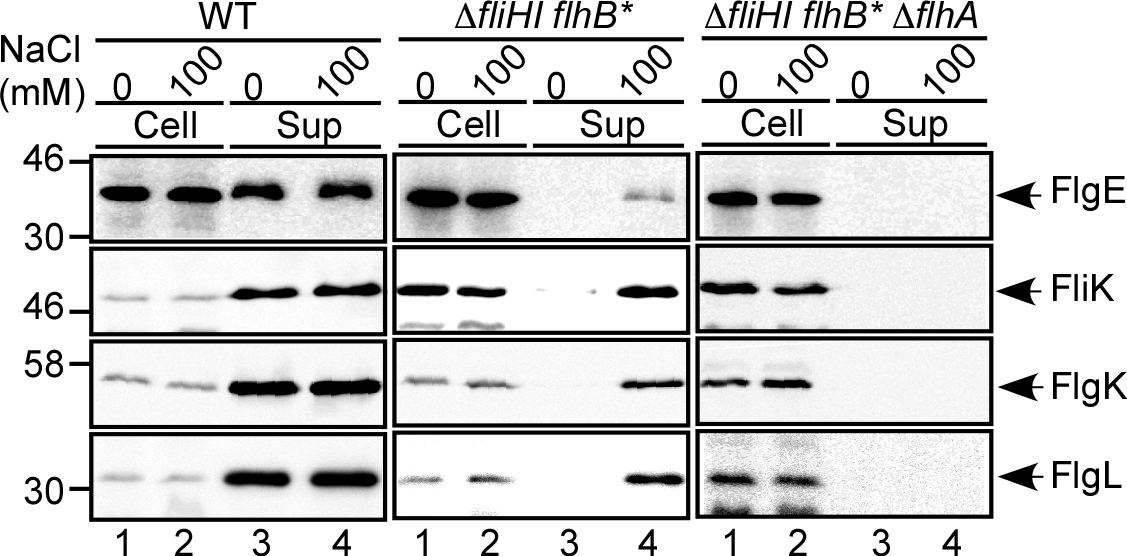

Supplement: S4 Fig — Immunoblotting, using polyclonal anti-FlgE (1st row), anti-FliK (2nd row), anti-FlgK (3rd row) or anti-FlgL (4th row) antibody, of whole cell proteins (Cell) and culture supernatant fractions (Sup) prepared from SJW1103 (WT), MMHI0117 (ΔfliHI flhB*) and NH004 (ΔfliHI flhB* ΔflhA) grown exponentially at 30°C in T-broth with or without 100 mM NaCl at external pH 7.5. (TIF) [file ppat.1005495.s004.tif]

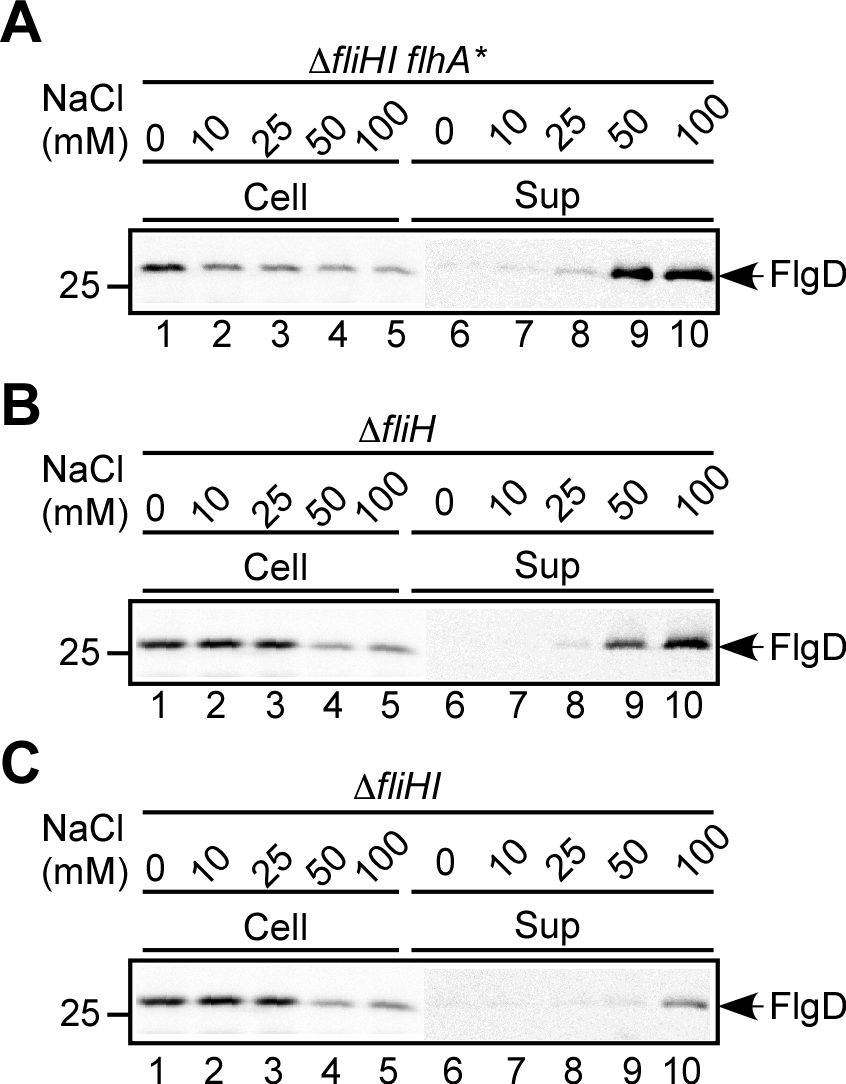

Supplement: S5 Fig — Immunoblotting, using polyclonal anti-FlgD antibody, of whole cell proteins (Cell) and culture supernatant fractions (Sup) prepared from (A) MMHI0132 (ΔfliHI flhA*), (B) MKM11 (ΔfliH), and (C) MMHI001 (ΔfliHI) grown at 30°C in T-broth containing 10 mM, 25 mM, 50 mM or 100 mM NaCl at external pH 7.5. (TIF) [file ppat.1005495.s005.tif]

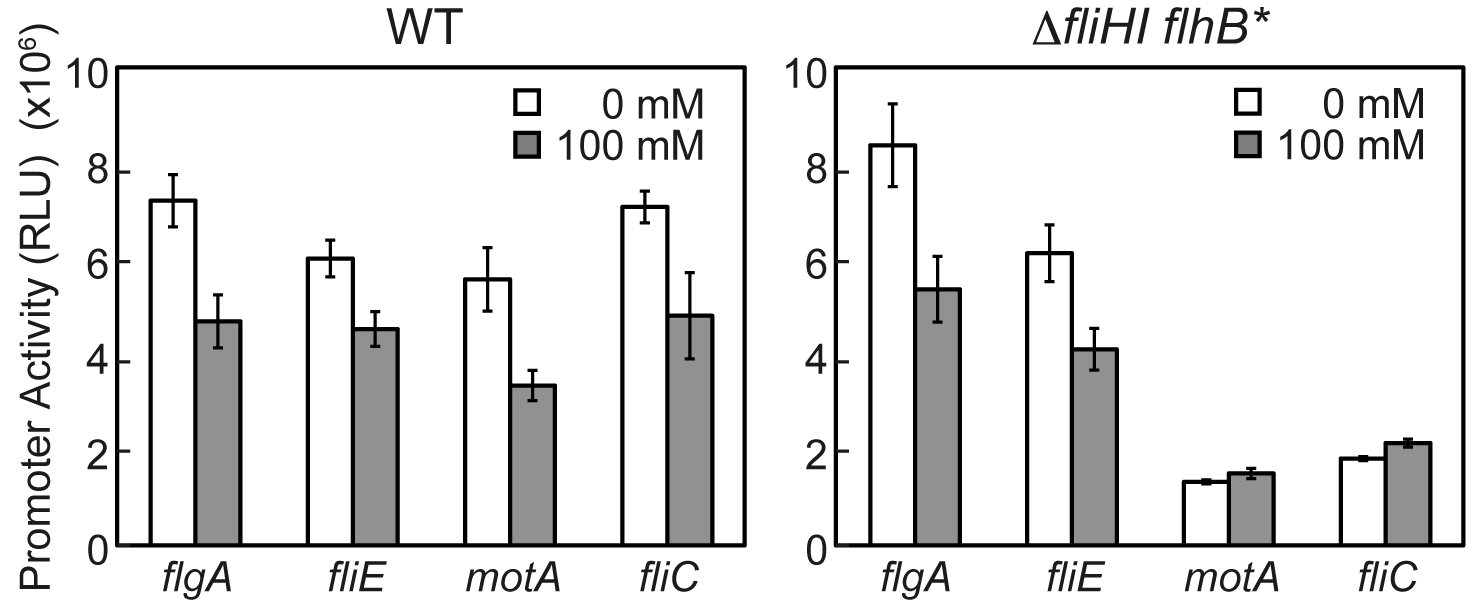

Supplement: S6 Fig — SJW1103 (WT) and MMHI0117 (ΔfliHI flhB*) were transformed with pRG19::cat (PmotA), pRG39::cat (PfliC), pRG51::cat (PflgA) or pRG19::cat (PfliE). Bioluminescence was measured as a promoter activity by a microplate reader. Vertical bars show standard deviations of four independent biological replicates. (TIF) [file ppat.1005495.s006.tif]

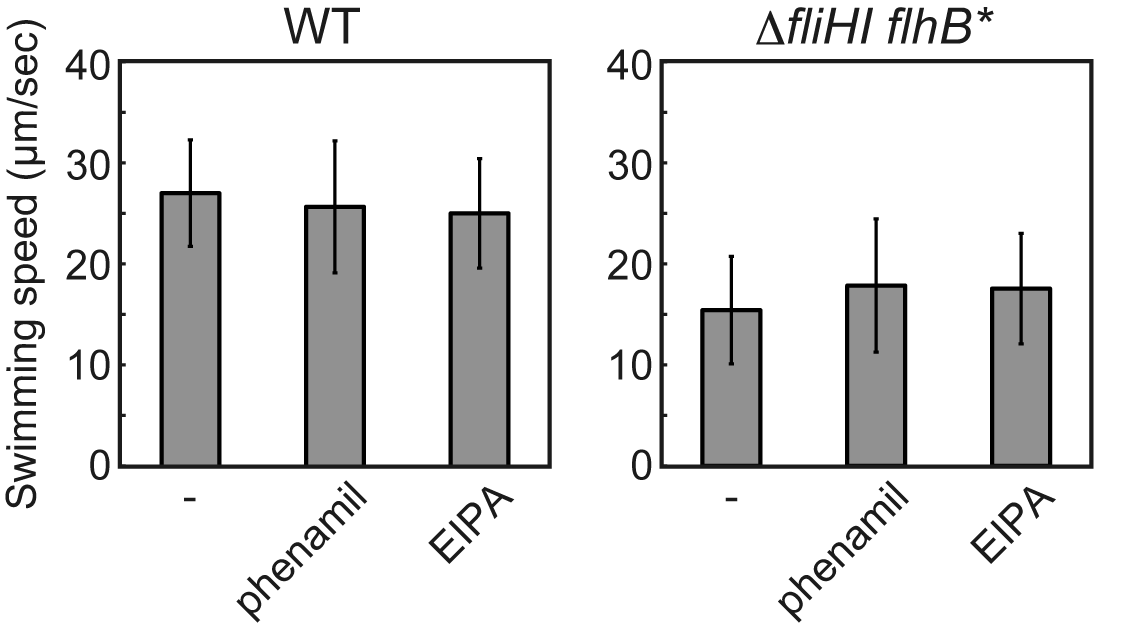

Supplement: S7 Fig — Swimming speeds of SJW1103 (WT) and MMHI0117 (ΔfliHI flhB*) were measured in T-broth containing 100 μM phenamil or 100 μM EIPA at an external pH of 7.5. More than 30 cells were measured. Vertical bars indicate standard deviations. (TIF) [file ppat.1005495.s007.tif]

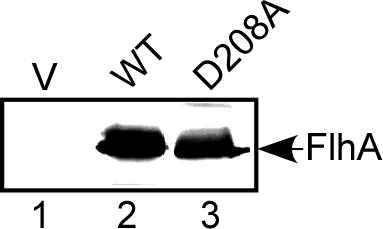

Supplement: S8 Fig — Immunoblotting, using polyclonal anti-FlhA antibody, of whole cell proteins prepared from SJW1368 carrying pBAD24 (V), pNH319 (WT) or pNH319(D208A). (TIF) [file ppat.1005495.s008.tif]
